# Nanostructures and Nanomaterials Integrated into Triboelectric Nanogenerators

**DOI:** 10.3390/mi16040403

**Published:** 2025-03-29

**Authors:** Shujie Yang, Victor Klinkov, Natalia Grozova, Svetlana Shalnova, Tatiana Larionova, Oleg Tolochko, Olga Klimova-Korsmik

**Affiliations:** 1 Department of Physics and Materials Technology, Institute of Machinery, Materials and Transport, Peter the Great St. Petersburg Polytechnic University, 195251 Saint Petersburg, Russia; yangshujie216@gmail.com (S.Y.); klinkovvictor@yandex.ru (V.K.); grozova_natalia@mail.ru (N.G.); larionova@hotmail.com (T.L.); tolochko_ov@spbstu.ru (O.T.); 2 World-Class Research Center “Advanced Digital Technologies”, State Marine Technical University, 190121 Saint Petersburg, Russia; s.shalnova@corp.smtu.ru

**Keywords:** triboelectric nanogenerator (TENG), nanostructures, nanomaterials

## Abstract

The pursuit of eco-friendly and renewable power generation has driven technological breakthroughs in nanoscale engineering, particularly regarding triboelectric nanogenerators (TENGs). These devices have become a focus of interest due to their capacity to effectively transform kinetic energy into electrical power via combined triboelectrification and electrostatic charge separation mechanisms. TENGs now find expanding implementations across multiple fields including in flexible electronics, autonomous sensing systems, and ambient energy conversion technologies. Enhancing TENG performance critically depends on the strategic design and application of nanostructures and nanomaterials. Nonetheless, challenges such as material selection, compatibility, homogeneous dispersion, interfacial stability, and production scalability must be overcome to advance TENG technology. Moreover, the mechanisms by which nanomaterials contribute to the triboelectric effect remain insufficiently understood, underscoring the necessity for systematic theoretical models. This review provides a comprehensive overview of recent advancements in integrating nanostructures and nanomaterials into TENGs, elucidating their roles, advantages, and underlying mechanisms in enhancing energy conversion efficiency, while identifying key challenges and proposing future research directions.

## 1. Introduction

With rapid industrial development, the demand for energy is increasing, promoting the development of energy acquisition technologies [1,2]. Within the spectrum of emerging energy solutions, triboelectric nanogenerators (TENGs) [3] have attracted significant research interest owing to their exceptional capability in transforming kinetic energy sources into electric power. These systems function through synergistic mechanisms combining surface charge transfer phenomena with induced electrostatic potential differences. Characterized by structural simplicity, economic feasibility, and superior energy transformation performance [4,5], such devices demonstrate consistent operational stability across diverse ambient parameters. Consequently, TENG-based energy harvesting has evolved into a viable approach for capturing biomechanical motions and industrial equipment oscillations, along with oceanic and atmospheric dynamic variations, subsequently generating usable electrical output. They can be effectively utilized in such applications as wearable devices, self-powered sensors, and others [6,7,8].

Enhancing charge density on surfaces has been identified as a critical factor in optimizing TENG operational efficacy [9,10]. This parameter is predominantly determined by the electron affinity disparity between contacted substances, making material selection a vital optimization strategy. Nanostructured architectures exhibit elevated surface-to-volume ratios that modulate contact electrification through alterations in work function characteristics and charge carrier concentrations. Incorporation of nanoscale additives into polymeric tribolayers represents an effective approach for enhancing TENG output characteristics. Such interfacial modifications promote intensified charge transfer events during material contact–separation cycles, thereby amplifying charge accumulation capacity. Furthermore, incorporating nanostructures into the triboelectric layer effectively enhances output by increasing the contact surface area relative to flat films [11]. Nanopillar-array architectures on polydimethylsiloxane (PDMS) have been demonstrated to significantly improve TENG performance by optimizing the contact area [12]. Moreover, reference [13] indicates that porous nanostructures in TENGs yield superior performance compared to flat surfaces under humid conditions. Consequently, polymer-based composites containing nanostructures or featuring nanostructured surfaces are of considerable interest for TENG applications.

Despite the promising potential of nanomaterials and nanotechnology in TENGs, several challenges persist in both research and practical applications. The properties of nanomaterials vary considerably; therefore, selecting materials with strong compatibility with the matrix is crucial when fabricating composite materials for TENGs [14]. Moreover, achieving uniform dispersion of nanomaterials remains an urgent challenge, as their aggregation within composites can cause property inconsistencies that adversely affect the power generation efficiency of TENGs.

**Figure 1:**
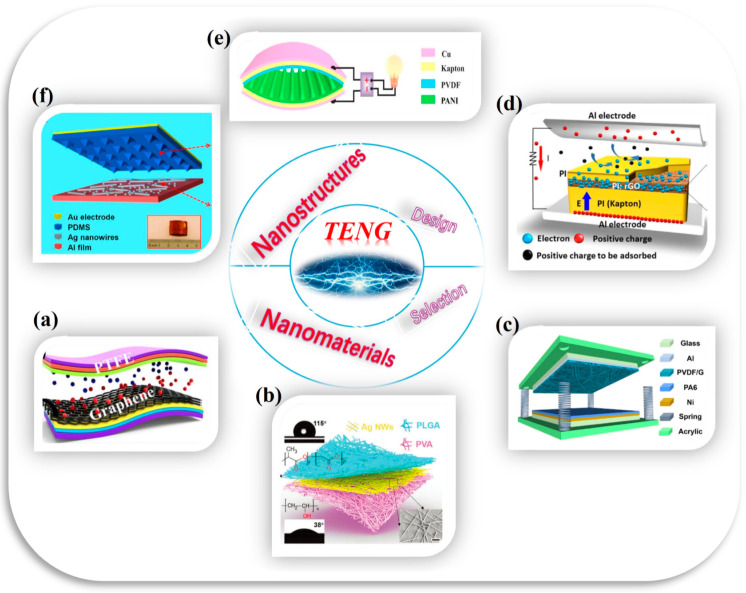
Structural representations of TENG configurations incorporating nanomaterial-enhanced architectures. (**a**) Flexible TENG architecture employing graphene dopants and Al_2_O_3_ nanoparticle integration [18] (Nanomaterials, 2021); (**b**) three-dimensional nanofiber network configuration in all-fiber TENG systems [19] (Science Advances, 2020); (**c**) three-axis rendered view of PVDF/G-PA6 composite-based TENG fabrication [20] (Nano Energy, 2021); (**d**) vertical contact–separation operational modality utilizing PI:rGO layered films [21] (Nano Energy, 2017); (**e**) design schematic of polyaniline-configured TENG construction [22] (Nano Energy, 2018); (**f**) TEAS device with dual-surface modifications via micropatterned PDMS geometries and Ag nanowire/nanoparticle composites [23] (ACS Nano, 2013).

From a theoretical standpoint, the mechanisms through which nanomaterials contribute to the triboelectric effect remain inadequately elucidated. The occurrence of the triboelectrification effect is closely related to the electron transitions between energy bands. Doping with nanomaterials can adjust the energy band structure of the material, allowing for more efficient attraction or release of electrons, thereby enhancing the triboelectrification effect. Although several studies have proposed models to describe the influence of nanomaterials on charge separation and transfer [15,16,17], the understanding of the underlying microscopic processes remains limited. Consequently, it is imperative to develop more systematic theoretical models to guide the design and optimization of nanomaterials for the advancement of TENG technology. These computational frameworks are poised to serve as indispensable tools for deciphering the operational mechanisms and optimizing the functional capabilities of mechanical-to-electrical energy conversion systems. Figure 1 Schematic diagram demonstrating the application of nanomaterials and nanostructures in the design of enhanced TENG outputs [18,19,20,21,22,23].

This review aims to provide a comprehensive summary of recent advancements in integrating nanostructures and nanomaterials into TENGs, with a focus on their roles, benefits, and mechanisms for enhancing energy conversion efficiency. Various nanostructure types are examined in terms of their specific contributions to TENG performance. In addition, the material selection process is analyzed, highlighting the advantages of nanomaterials such as carbon, metals, and metal oxides. This review elucidates the current state of the art, identifies key challenges, and proposes future research directions for the application of nanostructures and nanomaterials in TENGs.

## 2. Working Principle of Triboelectric Nanogenerators

### 2.1. Contact-Electrification

Contact electrification (CE) [24] describes the charge generation process occurring between dissimilar materials during interfacial contact–separation cycles. This triboelectric phenomenon, colloquially termed the ancient electricity effect, has persisted as a scientific enigma since its initial documentation over two millennia ago. While extensively documented, fundamental aspects of CE’s operational physics—especially regarding dominant charge carriers (electrons versus ions) and their transfer dynamics—continue to stimulate rigorous investigation and theoretical discourse within the scientific community.

Three primary charge transfer modalities have been identified during interfacial contact events between materials: electron migration, ion displacement, or charged material transfer [25]. Through Kelvin probe force microscopy analyses, investigations into CE’s fundamental nature have established electron migration predominance in solid-state interface interactions [26].

**Figure 2:**
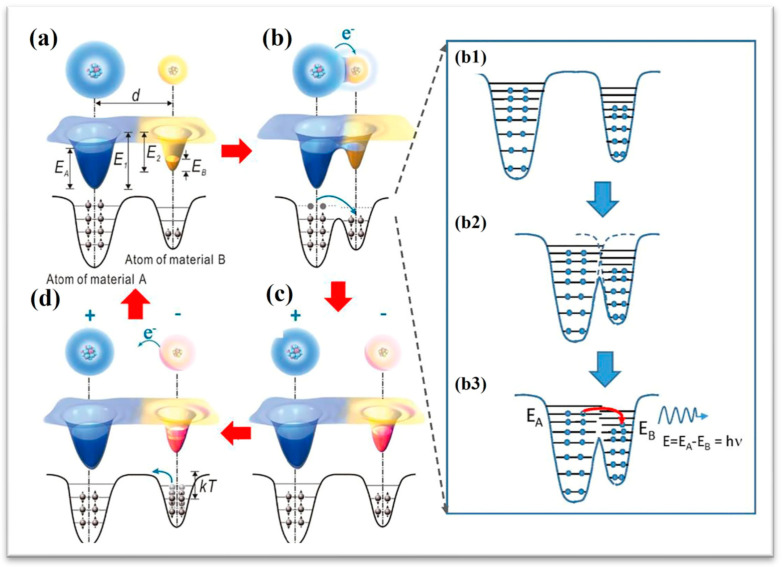
An electron-cloud–potential-well model proposed for explaining CE and charge transfer and release between two materials [17] (Advanced Materials, 2018). (**a**) Before contact; (**b**) after contact; (**b1**–**b3**) schematic diagrams of potential well changes while contacting; (**c**) separating; (**d**) after separation.

Prior to interfacial contact between material pairs A-B, electron occupation states within constituent atoms exhibit energy levels below the threshold required for surface electron emission (Figure 2a). Electron mobility between the constituents remains constrained by localized potential well confinement effects [17]. Upon interfacial contact, the original single-well potential configuration transforms into a symmetrical dual-well system, thereby lowering the activation barrier for electron tunneling. This quantum mechanical process enables cross-material electron transfer from donor atoms in substrate A to acceptor sites in substrate B (Figure 2b). Following contact termination, these translocated electrons persist as localized surface charges (Figure 2c). Pioneering work by Li [27] demonstrated atomically resolved photon emission signatures during solid-state CE processes, directly evidencing interfacial electron relocation events between atomic species of dissimilar materials during triboelectric interactions.

In contact electrification (CE) mechanisms, electron extraction potential emerges as a critical determinant. During triboelectric interactions, this parameter governs the energy threshold required for surface electron liberation. Substances exhibiting reduced electron extraction potentials demonstrate enhanced electron donation propensity, whereas counterparts with elevated values preferentially acquire electrons. Triboelectric material hierarchies are systematically categorized according to their respective electron extraction potentials. Such classifications enable predictive modeling of charge polarity distributions during interfacial contact events. Both the magnitude of tribocharge accumulation and its polarity directly correlate with the electron extraction potential differential between contacted surfaces [28]. Increased disparities in these surface energy parameters amplify charge separation efficacy, thereby optimizing the CE performance metrics. Surface engineering techniques including topological restructuring, roughness modulation, and composite material synthesis provide effective pathways for tuning electron extraction potentials.

### 2.2. Physical Mode of TENGs

**Figure 3:**
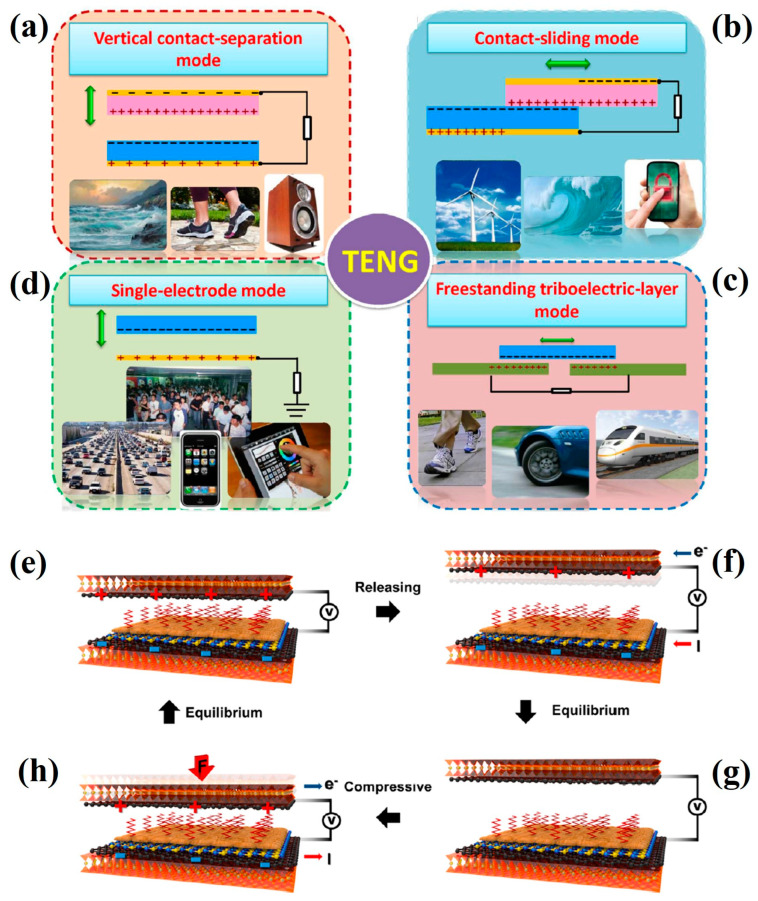
The four basic modes of TENG—(**a**) vertical contact–separation mode; (**b**) lateral sliding mode; (**c**) freestanding triboelectric-layer mode; (**d**) single-electrode mode [34]. (Royal Society of Chemistry, 2014). (**e**–**h**) Schematic power-generating mechanism of the non-contact triboelectric nanogenerators. (**e**–**f**): Releasing → release; (**h**–**g**): compressive → compression [35]. (American Chemical Society, 2022).

TENGs exhibit four primary operational configurations dictated by their working principles (Figure 3a–d): vertical contact–separation configuration, in-plane sliding mechanism, mono-electrode architecture, and independent triboactive layer design [6,29,30,31,32,33,34]. Surface charge polarization occurs between dissimilar materials through triboelectric interactions, creating complementary charge distributions. Mechanical separation of these materials spatially segregates these complementary charges, establishing an electrical potential gradient between the electrode components. This potential gradient drives directional electron transport via external conductive pathways. Cyclical alternation of interfacial contact states sustains periodic charge generation and recombination phenomena, ultimately producing continuous alternating current output.

The architectural configurations depicted in Figure 3a–d employ interfacial contact mechanics between triboactive surfaces. Nevertheless, the structural integrity and operational longevity of such devices face degradation from cyclic mechanical stresses and ambient operational conditions including temperature fluctuations and humidity variations. To address these constraints, innovative non-contact architectures (NC-TENGs) leveraging electrostatic field induction principles have been engineered [35,36,37]. Figure 3e–h schematically illustrate the working paradigm of these contactless systems. One friction layer surface is first injected with an electric charge, and when the two friction layers are close to each other (not in contact), the other friction layer surface generates an opposite charge under the electrostatic induction effect. During reciprocal motion between the triboactive interfaces, electrostatically induced charge accumulation establishes an electrical potential gradient. This energy differential drives directional electron migration across electrode pairs, yielding measurable current output.

## 3. The Theoretical Basis for Integrating Nanostructures and Nanomaterials into TENGs to Enhance Their Performance

The integration of nanostructures and nanomaterials into TENGs is theoretically grounded in several key principles, including increased surface area, enhanced charge density, optimized triboelectric properties, tailored energy band structures, and improved mechanical strength, among others. These advancements not only boost the performance of TENGs but also expand their potential applications in energy-harvesting technologies.

Nanostructures possess a high surface-to-volume ratio, which significantly increases the effective contact area between materials. This enhancement promotes greater charge generation during triboelectric interactions, resulting in higher output voltage and current. The larger surface area facilitates more efficient charge transfer and accumulation, which is critical for optimal TENG performance. Additionally, nanostructures can be fabricated using self-assembly techniques, allowing for precise control over their arrangement and orientation. This level of control can lead to optimized surface interactions and improved alignment with the triboelectric layers.

Nanomaterials can be engineered to optimize triboelectric properties effectively. By selecting materials with appropriate rankings in the triboelectric series, the efficiency of charge transfer can be maximized. For instance, combining different nanomaterials can create interfaces that exhibit enhanced triboelectric effects, thereby improving energy conversion efficiency. Additionally, nanomaterials can be designed with specific energy band structures to optimize electron mobility and enhance charge separation. This customization facilitates better electron transfer and enhances performance in energy conversion processes by minimizing the energy barriers for charge transfer. Furthermore, nanomaterials such as carbon nanotubes and graphene not only improve electrical properties but also increase the mechanical strength and durability of triboelectric nanogenerator (TENG) components. This leads to longer operational lifetimes and superior performance under mechanical stress, which are essential for wearable and flexible applications.

The operational current density of TENG systems is fundamentally governed by the surface charge concentration and dielectric characteristics of triboactive substances. Consequently, the selection of constituent materials critically determines a TENG’s operational efficacy, with the development and engineering of advanced triboelectric substances serving as the cornerstone for device performance enhancement [38].

Surface morphology engineering through micro/nanostructuring represents a pivotal strategy in TENG design. These topographical features effectively expand interfacial contact regions while amplifying charge density on triboactive surfaces [39]. The relative permittivity of triboactive substances directly governs the operational efficacy of TENG systems. Incorporation of high-permittivity nanofillers into these substances provides an effective pathway for permittivity augmentation.

## 4. Nanostructures Used in TENGs to Enhance Output Performance

Within research endeavors focused on TENG performance enhancement, surface engineering of triboelectric polymer layers has emerged as a foundational strategy for optimizing interfacial charge accumulation [40]. The application of laser etching, mold flipping processes, chemical etching and other techniques have introduced chemically eroded rough interfaces on friction surfaces, increasing the contact area between the friction layers.

### 4.1. Surface Nanopattern Structures

**Figure 4:**
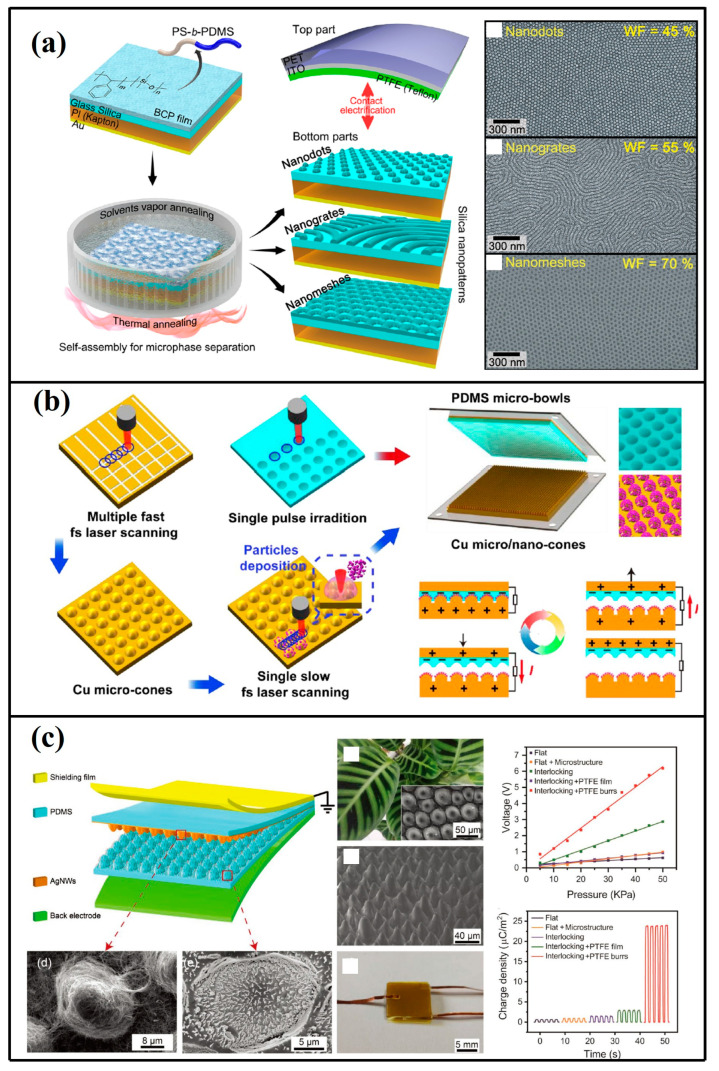
(**a**) Architectural layout detailing BCP-TENGs fabrication methodology [49] (Nano Letters, 2014). (**b**) Operational schematic illustrating manufacturing processes, structural components, and working principles of micro/nano-engineered triboelectric generators [50] (Elsevier Ltd., 2019). (**c**) Configuration diagram of TENG-based epidermal sensor architecture [51] (Advanced Functional Materials, 2020).

Nanoscale and microscale texturing of surface topographies has been demonstrated as a highly efficient approach for enhancing interfacial charge density [41,42,43,44,45,46,47,48]. A TENG featuring precisely engineered surface textures was developed by Chang Kyu Jeong [49] through block copolymer (BCP) self-assembly methodologies (Figure 4a). This fabrication technique enabled the creation of diverse silica nanoarchitectures such as quantum dot arrays, grating configurations, and mesh-like patterns using polystyrene-block-polydimethylsiloxane (PS-b-PDMS) thin film templates. The nano-engineered silica substrate was subsequently paired with Teflon as a complementary triboelectric surface. Comparative analysis revealed the optimized device generated an instantaneous current of ∼130 V with corresponding power output reaching ∼2.8 mA·m^−2^, representing 2.5-fold and 6.3-fold enhancements in voltage and current density, respectively, compared to non-textured tribolayer configurations.

Femtosecond laser processing was implemented by Ji Huang’s team [50] on dual-triboactive substrates comprising copper and PDMS. Laser ablation patterning generated directional microgrooves and conical protrusions on the copper substrate, while dual-scale concave architectures were engineered on the PDMS surface through controlled photothermal modification (Figure 4b). Quantitative analysis revealed the optimized configuration with copper microcones and PDMS microcavities achieved a surface charge density of 119.84 µC·m^−2^, demonstrating 4.58-fold enhancement compared to the non-engineered TENG counterparts. In [51], the cone-like interlocking microstructures on PDMS triboelectric layers were fabricated via the replication of the natural leaf surface. The upper PDMS layer was coated with silver nanowires and the lower PDMS layer was decorated with PTFE burrs, as shown in Figure 4c. Fabrication of the fine PTFE burrs arrays on the PDMS layer enabled the design to achieve a surface charge density of 23.98 µC/m^2^, which is about an order of magnitude higher compared with the TENG without surface modification.

**Figure 5:**
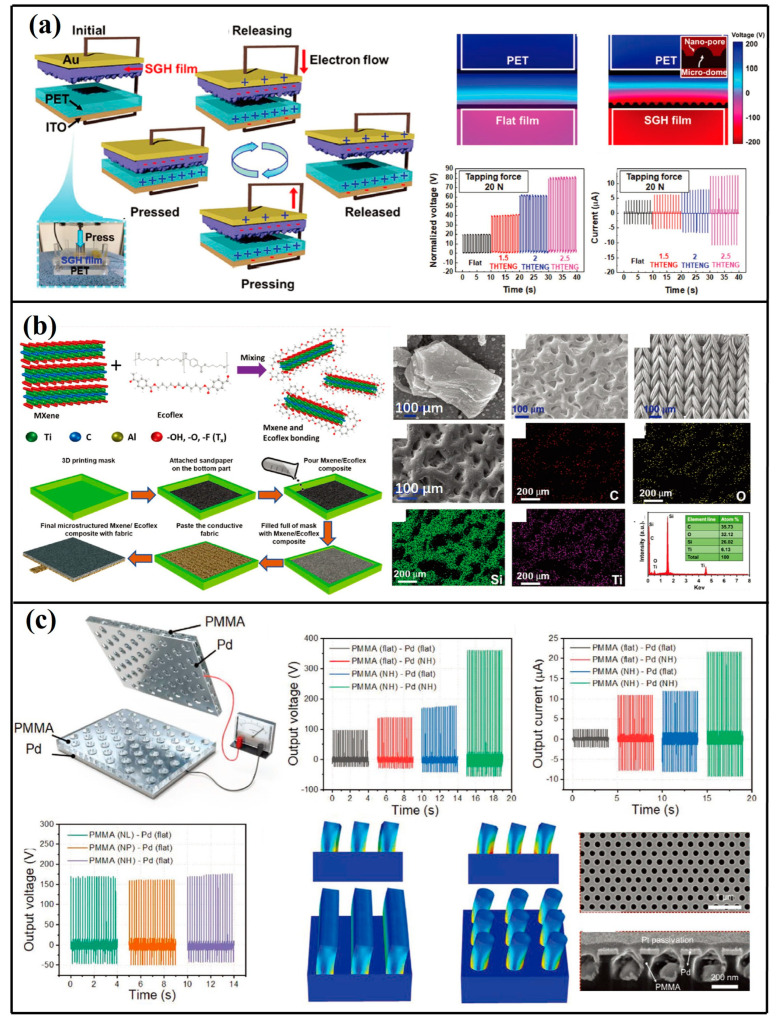
(**a**) Manufacturing workflow detailing EFS resin synthesis and textured glass–fabric-reinforced hybrimer (SGH) film production [52] (Advanced Functional Materials, 2020). (**b**) Schematic representation of interfacial bonding mechanisms between MXene nanosheets and Ecoflex elastomer [53] (Advanced Energy Materials, 2021). (**c**) Structural schematic of the architecturally refined TENG (AR-TENG) with scanning electron micrographs of triboactive interfaces [54] (Advanced Energy Materials, 2021).

Incorporation of hierarchical architectures onto triboactive surfaces has been demonstrated as an effective strategy for augmenting TENG operational efficacy. A research team led by Hyunh Wan Lee [52] engineered a siloxane-based composite film reinforced with textured glass fabric, featuring multiscale surface topography. Through synergistic application of molding techniques, roll-to-plate processing, and accelerated photochemical reactions, the composite achieved 210% surface area expansion relative to conventional configurations (Figure 5a). Device characterization revealed voltage outputs increasing from 40 V to 80 V, accompanied by current density enhancement from 4.3 µA to 12.5 µA. This performance enhancement correlates directly with the amplified charge storage capacity enabled by the engineered surface morphology.

MXene–polymer composite systems have been engineered with diverse nanostructural configurations to optimize triboelectric performance. A textile-integrated TENG was developed through integration of MXene/Ecoflex nanocomposites with nylon-functionalized textile electrodes [53]. Abrasive texturing utilizing variable-grit substrates generated microstructured surfaces on MXene/Ecoflex composites (Figure 5b). Surfaces textured with P600-grade abrasives demonstrated optimal electrical outputs. Ahn’s team [54] fabricated a PMMA-based TENG incorporating nanoporous architectures and palladium thin-film electrodes (Figure 5c). Nanoimprint lithography enabled precise engineering of PMMA surface morphologies including nanopillars (NP), nanolines (NL), and nanoholes (NH). While all the nanostructural variants enhanced energy conversion efficiency, NH configurations exhibited superior mechanical integrity under sustained mechanical loading. The NH-architected TENG achieved a power density of 1.547 W·m^−2^, generating 360 V peak voltage and 22 µA current while maintaining exceptional mechanochemical durability.

### 4.2. Surface Chemical Modification

**Figure 6:**
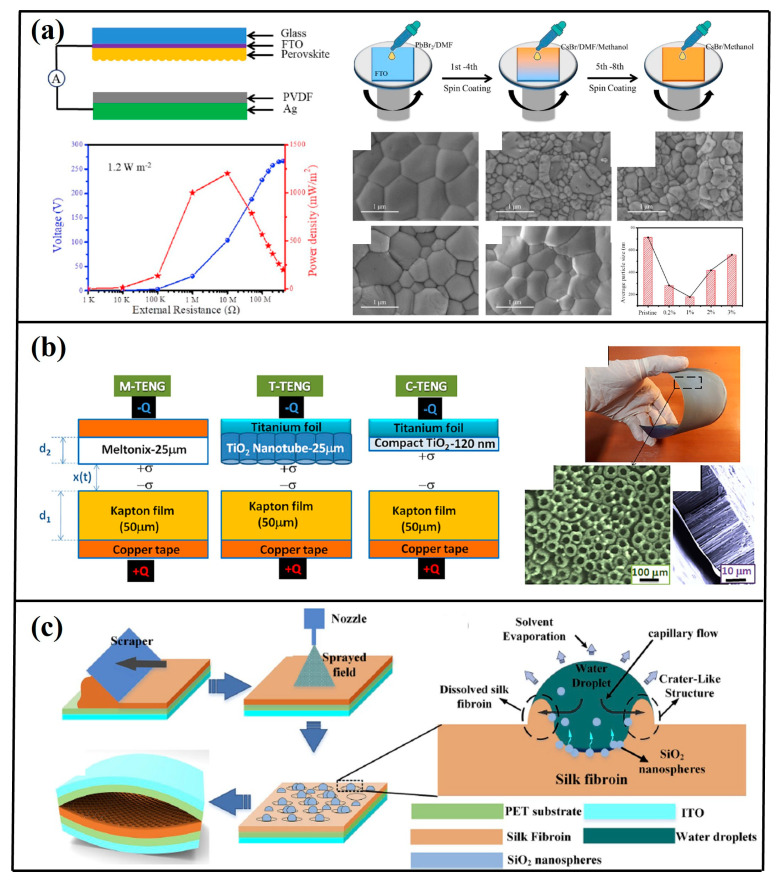
(**a**) Deposition schematic illustrating all-inorganic CsPbBr_3_; perovskite film fabrication via spin-coating methodology [56] (Elsevier Ltd., 2020). (**b**) Architectural layout detailing flexible TENG incorporating TiO_2_; nanotube arrays with corresponding nanostructural features [57] (Advanced Engineering Materials, 2018). (**c**) Manufacturing workflow for silk fibroin (SF)-integrated TENG ESE techniques [58] (Elsevier Ltd., 2020).

Surface chemical functionalization enables precise modulation of triboelectric polarity disparities between contact interfaces [55]. Etching-based surface engineering represents a prevalent methodology for augmenting charge accumulation capacity and operational efficiency in TENG systems. To optimize interfacial charge transfer dynamics (Figure 6a), morphology-tunable CsPbX_3_ (X = I, Br, Cl) perovskite architectures were synthesized via solvent-mediated etching protocols [56]. Comparative analysis with non-etched counterparts revealed enhanced crystalline growth regulation. The surface-modified CsPbBr_2.6_I_0.4_ configuration demonstrated peak performance metrics of 192 V open-circuit voltage and 16.7 μA short-circuit current, attributable to etching-enhanced surface charge density.

Anodic oxidation techniques were implemented by Raheleh [57] to fabricate self-aligned TiO_2_ nanotube arrays on triboactive substrates (Figure 6b). Charge density measurements revealed values of 110 nC·cycle^−1^ for nanostructured surfaces versus 15 nC·cycle^−1^ for planar analogues, accompanied by 40 V of output voltage and 1 μA·cm^−2^ current density. Electrohydrodynamic deposition processes were further employed to engineer micro/nano-textured silk fibroin films through aqueous spray-etching [58]. This approach yielded 260 V maximum output, corresponding to a 2.6-fold enhancement relative to non-textured configurations (Figure 6c).

In Section 4, the modification of friction layer surfaces through both physical and chemical methods were discussed. Following these modifications, the roughness of the friction layer surface is significantly improved, greatly enhancing the triboelectrification effect, increasing surface charge density, and ultimately boosting the performance of the friction nanogenerator. Table 1 shows information from all the literature cited in Section 4 about the TENGs based on surface modification. 

## 5. Nanomaterials Used in TENGs to Enhance Output Performance

### 5.1. Oxide Nanoparticle Doping Technology

The dielectric characteristics of triboactive interfaces with current density modulation are described here. Incorporation of high-permittivity nanofillers has been identified as an effective strategy for enhancing dielectric characteristics in polymeric films.

**Figure 7:**
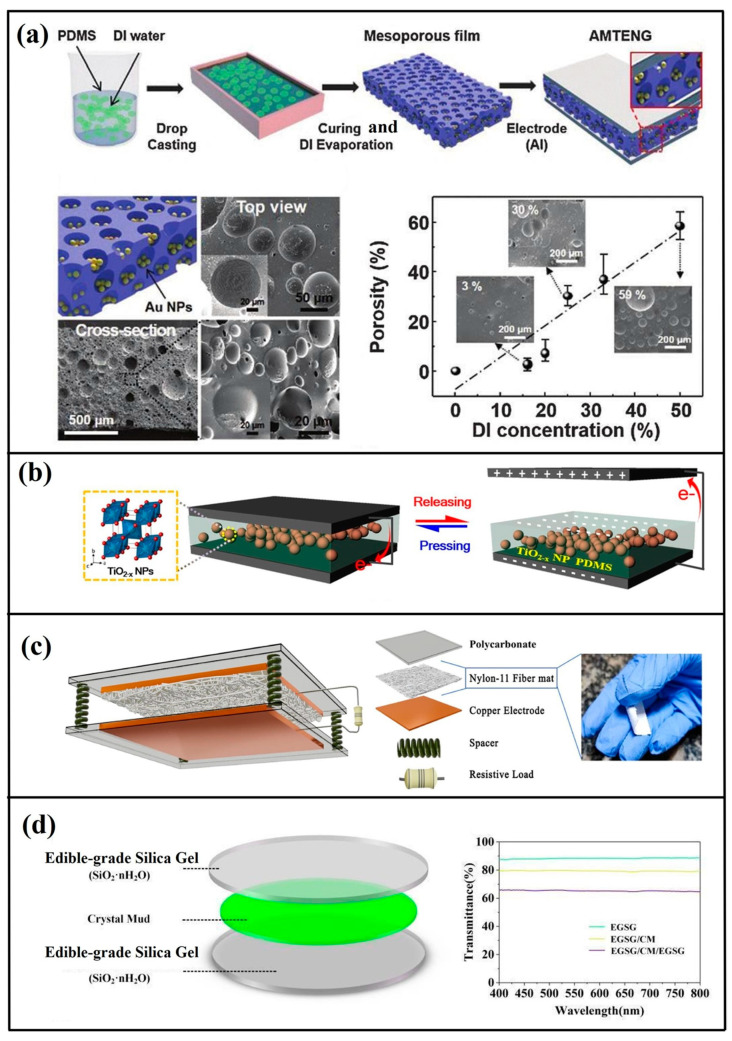
(**a**) Manufacturing workflow for dielectric–PDMS hybrid composites including sponge PDMS fabrication [59] (American Chemical Society, 2016). (**b**) Structural schematic of vertically operating TENG configuration with TiO_2_^−^x nanoparticle-embedded PDMS tribointerfaces, demonstrating periodic current generation through mechanical actuation [60] (Micromachines, 2018). (**c**) Device architecture of electrospun Nylon-11 nanofiber matrix-based TENG [61] (Elsevier Ltd., 2022). (**d**) Ultrapliant transparent EC-TENG architecture design [62] (Elsevier Ltd., 2021).

Prior research [59] has systematically investigated the coupled effects of permittivity and porosity on TENG electrical outputs. The tribonegative component comprised porous PDMS composites doped with nanostructured additives exhibiting distinct permittivity values: SiO_2_ (ε_r_ = 3), BaTiO_3_ (ε_r_ = 150), and SrTiO_3_ (ε_r_ = 300) (Figure 7a). Quantitative analysis revealed a strong permittivity-dependent enhancement in electrical outputs. Optimal porosity levels in the PDMS matrices exhibited proportional current density improvements, attributable to enhanced effective contact area-to-thickness ratios. The composite incorporating SrTiO_3_ (10% loading) achieved peak performance metrics of 19 nC·cm^−2^ charge density, 338 V open-circuit voltage, and 6.47 W·m^−2^ power density. Further optimization was demonstrated through TiO_2_^−^_x_ nanoparticle integration in PDMS matrices [60] (Figure 7b). Systematic evaluation of the filler concentrations (5–30 wt%) revealed maximal outputs (180 V, 8.15 μA) at 5 wt% TiO_2_ loading when paired with aluminum electrodes. Performance enhancements stemmed from synergistic dielectric constant elevation and oxygen vacancy modulation at polymer interfaces. Nylon-11/TiO_2_ nanocomposites were engineered via electrospinning techniques to optimize triboelectric responses [61] (Figure 7c). TiO_2_ incorporation (5 wt%) induced fiber diameter reduction (234 → 187 nm) while improving matrix integrity, yielding hydrophobic and antimicrobial properties. The optimized configuration generated 56 V and 5.1 μA.

Pioneering work by Kequan Xia [62] introduced an edible-grade transparent TENG utilizing silica gel/crystal clay composites (Figure 7d). The EC-TENG achieved record outputs of 25.96 μA short-circuit current, 1400 V open-circuit voltage, and 7.84 mW power, demonstrating dual functionality as self-powered biosensors and biomechanical energy harvesters.

Table 2 shows information about the TENGs using the high-dielectric nanoparticle doping method from all the literature cited in Section 5.1.

### 5.2. Application of Carbon Nanomaterials

Carbon nanomaterials play a crucial role in the design and enhancement of TENGs due to their diverse structures and properties. 

**Figure 8:**
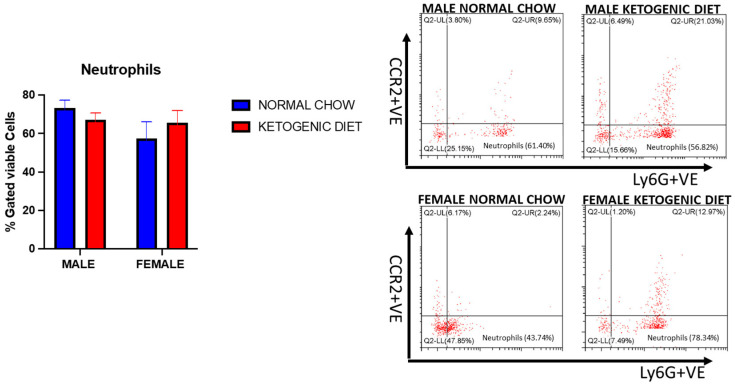
(**a**) Architectural configuration of AC@PVDF-based wearable triboelectric systems with enhanced relative permittivity [64] (American Chemical Society, 2020). (**b**) Operational schematic detailing multipurpose sensing devices employing TENG mechanisms [65] (Science Advances, 2020). (**c**) Functional schematic delineating Siloxene/Ecoflex charge generation stratum, LIG-integrated MoS_2_ charge confinement interface, and copper charge harvesting electrodes [63] (Advanced Functional Materials, 2022).

A novel TENG architecture integrating Siloxene/Ecoflex nanocomposites with LIG components was engineered by Kumar Shrestha [63], as depicted in Figure 8a. This configuration employed MoS_2_-functionalized LIG as a charge-trapping interfacial layer, effectively amplifying surface potential gradients to optimize device output characteristics. The engineered system additionally demonstrated superior humidity-responsive operational capabilities.

A TENG designed for biomechanical energy harvesting and active sensing was fabricated using AC particles, as shown in Figure 8b [64]. The AC particles were incorporated into a PVDF film, which increased its relative permittivity. Additionally, the specific microstructure of the AC particles provided a larger surface area for triboelectrification. Compared to a pure PVDF-based TENG, the TENG based on 0.8% AC@PVDF exhibited Voc, I_sc_, and power outputs that were 2.5, 3.5, and 9.8 times higher, respectively. Various types of graphene have also been utilized in TENGs. In reference [65], graphene was incorporated into PDMS to influence the dielectric permittivity of the friction materials. The TENG fabricated with a graphene/PDMS sponge (Figure 8c) demonstrated excellent output performance and high pressure-sensing sensitivity, making it suitable for use as a sensor.

**Figure 9:**
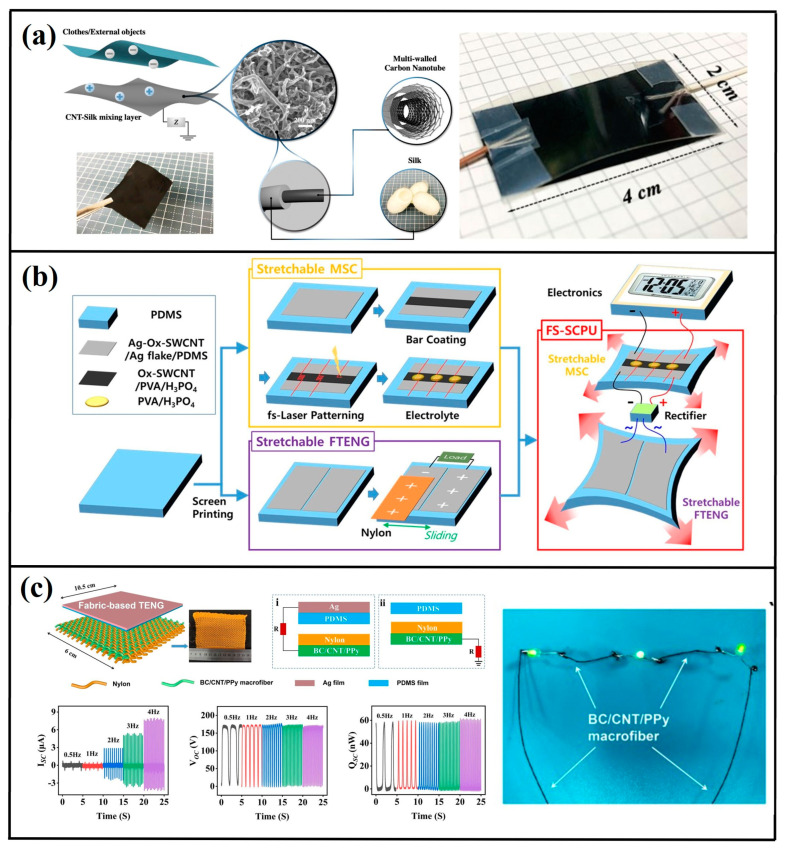
(**a**) Structural configuration of CNT–silk composite matrix-integrated triboelectric system [66] (Springer, 2022). (**b**) Manufacturing workflow for flexible self-sustaining power units (FS-SCPU) incorporating stretchable microsupercapacitors (MSC) and fiber-based triboelectric generators (FTENG), where alternating current is rectified to unidirectional flow via external circuitry [67] (Elsevier Ltd., 2021). (**c**) Functional schematic of textile-integrated triboelectric architecture with operational metrics [68] (Springer, 2022).

Carbon-based architectures, particularly CNT-enhanced systems, have become pivotal in triboelectric energy harvesting due to their structural uniqueness. Meng Su [66] engineered a minimalist triboelectric system capturing biomechanical energy from human locomotion (Figure 9a). This configuration synergized silk tribolayers with CNT-based electrodes, demonstrating peak electrical outputs of 8.73 μA short-circuit current, 262 V open-circuit voltage, and 285.91 μW·cm^−2^ power density while maintaining operational stability. Hye Jin Yang [67] fabricated a fully stretchable energy harvester utilizing oxidized SWCNT/polymer electrodes (Figure 9b). The device achieved 84.4 mW·m^−2^ instantaneous power density under 40% tensile deformation. Sanming Hu [68] pioneered sustainable TENG technology integrating CNT-reinforced cellulose macrofibers (Figure 9c), where nanomaterial incorporation significantly enhanced mechanical durability without compromising environmental compatibility. The reported TENG with a maximum I_sc_ of 0.8 μA, V_oc_ of 170 V, and output power of 352 μW/cm^2^ can work as self-powered sensor to monitor human motions.

Table 3 shows information about the TENGs containing carbon nanomaterials which were mentioned in Section 5.2.

## 6. Summary and Prospects

Recent research has achieved notable advancements in enhancing the output performance of TENGs through the incorporation of various nanostructures into their design. Incorporating micro/nanostructures, such as cones, stripes, pores, burr arrays, and biomimetic designs, on the friction layer surface of TENGs has proven highly effective. These complex geometries maximize the contact area between triboelectric materials, thereby improving charge generation via enhanced triboelectric effects. By strategically designing these nanostructures, researchers have considerably increased the charge density on the TENG surface, which is essential for boosting overall electrical output. This innovative approach not only optimizes TENG performance but also facilitates the development of more efficient energy-harvesting devices.

In addition to nanostructures, the integration of advanced nanomaterials has further enhanced TENG performance. Oxide nanoparticles such as SiO_2_, TiO_2_, BaTiO_3_, SrTiO_3_, and MnO_2_, along with carbon-based nanomaterials such as graphene and carbon nanotubes, have been employed to optimize TENG designs. These materials exhibit superior dielectric properties that improve energy storage and minimize dielectric losses during operation. The combination of high dielectric constants and enhanced conductivity not only improves energy conversion efficiency but also ensures TENG functionality under various operational conditions. By leveraging the unique properties of these nanomaterials, researchers have effectively increased the charge density on the TENG surface, leading to a marked enhancement in output performance. The synergy between nanostructures and nanomaterials represents a promising avenue for the development of self-powered devices and sustainable energy solutions.

However, the output power of TENGs remains insufficient, considerably limiting their practical applications. The energy conversion efficiency of TENGs is a crucial factor that must be considered. Currently, much of the research focuses on improving the output performance of TENGs, often overlooking their efficiency in converting mechanical energy to electrical energy. Therefore, calculating and enhancing the energy conversion efficiency of TENGs is a key research target. The precise energy analysis model presented by Zheng [69] offers valuable guidance in this area. Although the incorporation of various micro/nanostructures and materials enhances surface charge density, it is imperative to consider the operational environment. The friction layer must exhibit adequate wear resistance, and the overall TENG structure must retain stability.

While we previously noted that the incorporation of nanomaterials and nanostructures into TENGs significantly enhances their output performance, it is also important to consider the challenges and limitations associated with these processes. The uneven distribution of nanomaterials within the friction layer can adversely affect TENG performance. Additionally, handling and processing these nanomaterials often require specialized techniques and equipment, which adds complexity to the manufacturing process. Furthermore, high-performance nanomaterials tend to be expensive, potentially increasing the overall cost of TENG fabrication. The preparation of nanostructures on the surface of the friction layer typically involves intricate techniques and equipment, further complicating and raising the costs of TENG production. By overcoming the challenges associated with nanomaterial processing and stability, researchers can significantly improve the efficiency and applicability of TENGs in various fields.

From the perspective of material science, modifying the friction layer material used in TENGs is an effective approach to improving their output performance. However, considering that TENGs function as electronic devices that convert mechanical energy into electrical energy, optimizing the structural design and circuit management of TENGs [70] also plays a crucial role in enhancing their overall performance. In the next phase of TENG development, simultaneous improvements in both power output and structural robustness are essential.

## Figures and Tables

**Table 1:** Overview of the morphology of TENGs based on surface modification.

Surface Morphology of Modification	Max Voc	Max Isc	Max Power Density	Ref.
Nanodots, nanogrids, and nanoclusters	130 V	2.24 µA	122.8 mW/m^2^	[49]
Stripe and cone-like nanostructures	22.04 V	2.6 µA	210 mW/m^2^	[50]
Interlocking microstructures	6.2 V	26.29 nA	-	[51]
Textured hybrimer film	210 V	24 µA	824 mW/m^2^	[52]
MXene/Ecoflex rough microstructures	790 V	183 µA	9.5 W/m^2^	[53]
Nanohole pattern	360 V	22 µA	1.547 W/m^2^	[54]
Fine crystal structure	192 V	16.7 µA	1.2 W/m^2^	[56]
TiO_2_ nanotube	40 V	1 µA	17 mW/m^2^	[57]
Porous and hierarchically structures	260 V	6.7 µA	161.5 μW/cm^2^	[58]

**Table 2:** Overview of nanoparticles used in TENGs.

Nanoparticles	Max V_oc_	Max I_sc_	Max Power Density	Ref.
SiO_2_, TiO_2_, BaTiO_3_, and SrTiO_3_	338 V	36.24 µA	6.47 W/m^2^	[59]
TiO_2_-x nanoparticles	790 V	183 µA	9.5 W/m^2^	[60]
TiO_2_ nanoparticles	283 V	53 µA	1.02 W/m^2^	[61]
Silica gel and crystal clay	1400 V	25.96 µA	1.7 W/m^2^	[62]

**Table 3:** Overview of carbon nanomaterials used in TENGs.

Carbon Nanomaterials	Max Voc	Max Isc	Max Power Density	Ref.
Active carbon	157.06 V	20.47 µA	1.6 W/m^2^	[64]
Graphene	8 V	12.5 mA	-	[65]
Laser-induced graphene	31 V	0.63 µA	4.7 mW/m^2^	[63]
Carbon nanotubes	262 V	8.73 µA	2.85 W/m^2^	[66]
Oxidized carbon nanotubes	90 V	6.3 µA	84.4 mW/m^2^	[67]
Carbon nanotubes	170 V	0.8 µA	3.52 W/m^2^	[68]
